# Sunflower-Assisted Bio-Derived ZnO-NPs as an Efficient Nanocatalyst for the Synthesis of Novel Quinazolines with Highly Antioxidant Activities

**DOI:** 10.3390/antiox11040688

**Published:** 2022-03-31

**Authors:** Mahesh S., Boya Palajonnala Narasaiah, Himabindu B., Balaji G. L., Jangampalli Adi Pradeepkiran, Harihara Padhy

**Affiliations:** 1 PG&Research Department of Chemistry, Thanthai Hans Roever Collage (Autonomous), Affiliated to Bharathidasan University, Perambalur 621220, India; maheshreddysm@gmail.com; 2 Department of Physics, Indian Institute of Technology (IIT), Tirupati 517506, India; narsipalajonna@gmail.com; 3 Department of Zoology, Sri Venkateswara University, Tirupati 517502, India; himabindu.bt@gmail.com; 4 Department of Chemistry, School of Advance Science and Languages, VIT Bhopal University, Bhopal 466114, India; harihara.padhy@vitbhopal.ac.in; 5 Department of Internal Medicine, Texas Tech University of Health Science Centre, Lubbock, TX 79415, USA; 6 Department of Chemistry, GITAM Institute of Science, GITAM (Deemed to be University), Visakapatnam 530045, India

**Keywords:** green synthesis, ZnO-NPs, 1,2-dihydro quinazolines, antioxidant activity

## Abstract

The present report presents a green method for the rapid biogenic synthesis of nanoparticles that offers several advantages over the current chemical and physical procedures. It is easy and fast, eco-friendly, and does not involve any precious elements, hazardous chemicals, or harmful solvents. The synthesized ZnO nanoparticles were characterized using different techniques, such as UV-Visible spectroscopy. The surface plasmon resonance confirmed the formation of ZnO nanoparticles at 344 nm, using UV-Visible spectroscopy. The leaf extract acts as a source of phytochemicals and is primarily used for the reduction and then the formation of stable ZnO nanoparticles by the characteristic functional groups of the extract; the synthesized ZnO nanoparticles were identified using FTIR spectroscopy. The crystalline nature of ZnO-NPs was confirmed via powder X-ray diffraction (XRD). Size and morphology were measured via high resolution transmission electron microscopy (HR-TEM) analysis. The stability of the nanoparticles is established using dynamic light scattering (DLS) and thermogravimetric analysis (TGA). The synthesized ZnO nanoparticles have been found to be a good and efficient catalyst for the synthesis of novel 1,2-dihydro quinazoline derivatives under the green method via a one-pot reaction of 2-amino benzophenone, 1,3-diphenyl-1H-pyrazole carbaldehydes, and ammonium acetate. The synthesized compounds (**4a**–**o**) were characterized by the ^1^H NMR, ^13^C NMR, and HRMS spectra and were further validated for free-radical scavenging activity. The synthesized ZnO nanoparticles exhibited good antioxidant activity.

## 1. Introduction

Nanotechnology is a fast-emerging area of science that has a diverse range of applications, such as in medicine and industry. A nanoparticle is a small particle that ranges between 1 and 100 nm in size. It acts as a link between the macroscopic and microscopic atomic worlds [1,2,3]. Today, many technologies are making quantum leaps in the field of medicine, industries, catalysis, and environmental cleansing using nanoparticles [4,5]. There are several methods for synthesizing nanoparticles, including sol-gel, pyrolysis, hydrothermal synthetic methods, etc. In addition, some physical and chemical methods are widely used by researchers to synthesize nanoparticles. The preparation of nanoparticles using biosynthetic methods has received much attention in recent years, in the search for a non-toxic, eco-friendly, low cost, and safe unwanted wastewater particle remover. Nanoparticles produced by plants are highly stable, differing in shape and size when compared with other chemical methods employing toxic chemicals, additives, or capping agents [6,7]. Nanoparticles have been fabricated using chemical synthesis routes under specific conditions. During the precipitation process from liquid phases, surface controlling agents have been added after the formation of precipitates. Green chemistry offers an ideal method for the clean, reliable, biocompatible, and eco-friendly preparation of nanoparticles [8,9], wherein the green catalyst is used for organic transformation processes like bond formation [10]. The biological synthesis of metal oxide NPs using plant extracts offers an alternative to chemical methods for reducing the amount of hazardous and toxic chemicals in the environment, as well as for synthesized metal oxide nanoparticles with well-defined size and shape [11,12]. Different nanoparticles are currently being used in various areas. Among these different nanoparticles, transition-metal nanoparticles have a wide application across many fields, including chemistry, physics, biotechnology, and environmental chemistry [13,14]. In particular, transition-metal nanoparticles have been used in microbial studies, e.g., silver compounds have been used to treat burns and wound infections [15]. Various salts from silver and their derivatives are currently used as antimicrobial agents [16,17,18]. The current study represents the first time that the green patch approach has been reported regarding the biosynthesis of ZnO nanoparticles from a sunflower-leaf aqueous extract. The catalytic properties of ZnO nanoparticles were investigated in organic applications. This investigation indicates that ZnO nanoparticles, prepared by green synthesis, can be used efficiently and are highly active as a catalytic agent.

**Figure 1:**
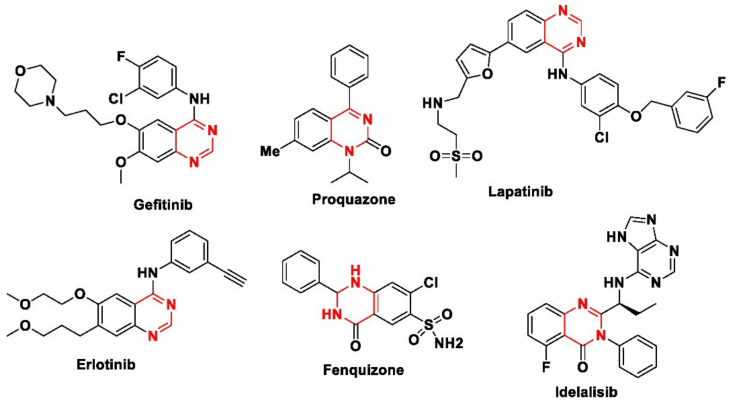
Biologically active quinazoline derivatives.

In recent years, the synthesis of six-membered quinazoline heterocycles and their derivatives (Figure 1) has become attractive in synthetic organic chemistry, due to their wide-ranging biological and medicinal properties [19,20], such as their antibacterial [21], anticarcinogenic [22], antiviral [23], antitubercular [24], anticancer [25,26], anti-convulsant [27], antiarthritic [28], anti-inflammatory [29,30], anti- fungal [31] and anti-diabetes [32] activities.

In addition, quinazolines are commonly found as the building blocks for a wide variety of natural products, such as alkaloids, and in various other microorganisms, including *Bouchardatia neurococca*, *Peganum nigellastrum*, *Bacillus cereus*, and *Dichroa febrifuga*. Usually, the traditional synthesis of quinazolines involves the reactions of Bischler cyclization, dicarbonyl compounds with diamines, and anthranilic acids with amide [33]. However, a few examples regarding the synthesis of 1,2-dihydroquinazolines have been reported in the literature. The reaction of 2-aminobenzamide with benzaldehyde [34,35,36], 2-aminobenzophenone with benzylic amines [37,38], 2-aminobenzophenone with benzaldehyde [39,40], 2-aminobenzophenone with isatin [41], 2-aminobenzophenone with methylarenes [42] and 2-aminobenzophenone with urea [43] represent numerous methods for the preparation of 1,2-dihydroquinazolines. However, these methods suffer from many limitations, such as harsh reaction conditions, expensive reagents, difficult purification procedures, lesser product yields, and metal catalysts, as well as a more than stoichiometric amount of reagents. Sustainable chemistry has increased in importance recently due to its environmental compatibility. Therefore, the development of facile, mild, and environmentally benign recyclable synthetic protocols for functionalized 1,2-dihydroquinazoline derivatives is highly desirable. Chaudhari et al. [44] have reported an efficient and recyclable heterogeneous CeO_2_-Pt nanocatalyst in the synthesis of 2-substituted quinazolines under reflux conditions. Karnakar et al. [45] reported an efficient route to the synthesis of quinazolines by using recyclable graphite oxide; this route was found to be more efficient than the other investigated methods. Based on the literature survey and the importance of the 1,2-dihydroquinazolines, an eco-friendly and efficient one-pot procedure for the synthesis of 2-(1,3-diphenyl-1H-pyrazol-4-yl)-4-phenyl-1,2-dihydroquinazolines via the reaction of 1,3-diphenyl-1H-pyrazole-4-carbaldehydes, 2-aminobenzophenone, and ammonium acetate in a green method, using ZnO nanoparticles as a catalyst, has been developed.

## 2. Materials & Methods

### 2.1. Materials

Fresh sunflower leaves were collected in the village of Chilakaladona in the Tungabhadra River area, Andhra Pradesh, India. Zinc acetate dihydrate Zn(O_2_CCH_3_)_2_(H_2_O)_2_, organic solvents were purchased from Sigma-Aldrich, Karnataka, India. All reagents of AR grade were used without purification. Distilled water was used as a solvent throughout the process. All the chemicals and solvents used for the preparation of 1,2 dihydroquinazoline derivatives were procured from Alfa Aesar and Spectrochem (Haverhill, MA, USA). Thin-layer chromatography was used for the completion of the reactions. Dried and calibrated glassware was used for carrying out the reactions. The ^1^H NMR and ^13^C NMR values were analyzed using a Bruker Advance 400 MHz spectrometer (Billerica, MA, USA) in deuterated chloroform (CDCl_3_); we used tetramethyl silane (TMS) as the internal standard. The chemical shift values of the synthesized compounds were expressed in parts per million (ppm). An Elchem digital apparatus (Oslo, Norway) with a capillary tube was used to determine the melting points. The percentage of inhibition of the samples in the antioxidant studies was measured with a Shimadzu UV-Visible spectrometer (Kyoto, Japan). The HRMS for the synthesized compounds were analyzed with a SCIEX Zeno (Framingham, MA, USA). The TOF and FTIR analyses of the ZnO nanoparticle were carried out using a Hitachi spectrophotometer (Tokyo, Japan).

### 2.2. Collecting Plant Material and Preparation of the Leaf Extract

Sunflower leaves were collected from an agricultural sunflower field. The collected leaves were washed with treated water; after washing thoroughly, they were then dried at room temperature overnight. The dried sunflower leaves were powdered using a conventional grinder and sieved through a 100mesh standard sieve. About 3 g of the ground leaves were added to 100 mL of double-distilled water, then they were heated on a hot plate at 80 °C for 50 min. After 50 min, a color change was observed due to the phytochemicals having been dissolved in the double-distilled water. After cooling, the extract was filtered using a Whatman No.1 filter paper (Maidstone, UK), then the extract was stored at 4 °C until later use with the preparations of ZnO nanoparticles.

### 2.3. Preparation of ZnO-NPs Using Sunflower Leaf Extract

**Figure 2:**
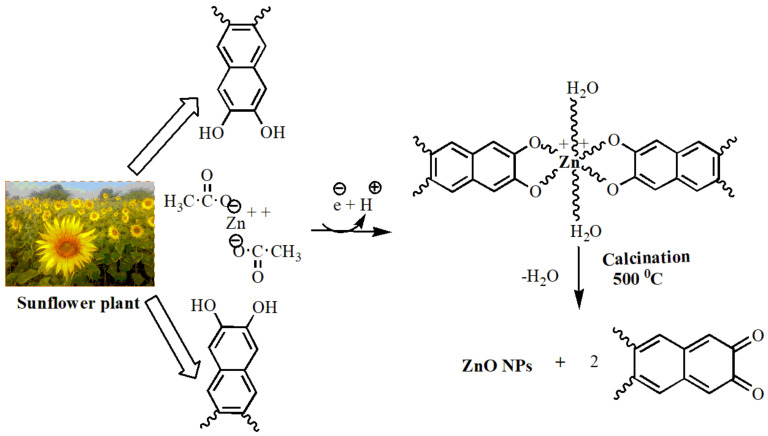
A possible mechanism for the synthesis of ZnO NPs using sunflower-leaf aqueous extract.

About 0.05 M Zn(O_2_CCH_3_)_2_(H_2_O)_2_ solution (30 mL) was dissolved in 30 mL of the plant extract and stirred using a magnetic stirrer at 500 rpm for 3 h at 80 °C. The colorless fluid of the zinc acetate solution changed to a yellowish milky white color with the addition of the sunflower leaf extract. After 3 h, a white precipitate developed when colored nanoparticles were formed, which indicated the formation of ZnO nanoparticles. The solution was then carefully transferred and centrifuged for 30 min at 3500 rpm. The resulting pellet was then washed twice with absolute alcohol and evaporated, followed by double-distilled water cleaning to remove any biomolecules and impurities, and was then centrifuged again under the same conditions The pure, fine pellets were collected and dried in a hot-air oven, then crushed into powder for further analysis. The synthesized sample was then subjected to calcination in a muffle furnace, annealing at 500 °C for 3 h. The crystalline nature of the pure ZnO nanoparticles is shown in Figure 2.

### 2.4. Characterization of Synthesized ZnO NPs

The optical properties of ZnO nanoparticles were characterized based on their absorption band, using UV-Visible spectroscopy. The synthesized ZnO nanoparticles and sunflower leaf extract with a dye degradation solution were monitored using a UV/Vis spectrophotometer (Shimadzu, model: UV-2450) with an absorption range from 200 to 800 nm. The identification of functional groups in the leaf extract and ZnO nanoparticles was performed using FTIR with a Shimadzu IR AFFINITY-1. The crystal structure of the synthesized ZnO nanoparticles was studied using XRD (D8 Advance diffractometer, Bruker, Mannheim, Germany). The morphology and size of the synthesized ZnO nanoparticles were visualized by placing a drop of the sonicated sample, well dispersed, on the Cu grid, and using high-resolution transmission electron microscopy (HR-TEM) (Model: JEOL JEM 2100, Peabody, MA, USA) at a resolution of 0.1 nm, with an acceleration voltage of 200 kV. The TEM also provided additional information. The elemental composition of ZnO was analyzed using energy-dispersive X-ray (EDAX) analysis. The electrophoretic mobility of the samples was analyzed with a Horiba Scientific (SZ-100) nanoparticle instrument (Kyoto, Japan). Dynamic light scattering (DLS) was used to examine the stability of the nanoparticles, based on the charge distribution and the thermal stability of the ZnO nanoparticles, analyzed by thermogravimetric (TGA) analysis (2100F with an acceleration voltage of 100; KV-TGA-JOEL).

### 2.5. General Procedure for the Synthesis of 1,2 Dihydroquinazoline Derivatives (***4a***–***o***)

A mixture of 2-amino-5-chlorobenzophenone (**1**) (1 mmol), 1,3-diphenyl-1H-pyrazole-4-carbaldehyde (**2**) (1 mmol), ammonium acetate (**3**) (3 mmol), and ZnO nanoparticles (10 mol%) (Table 1) in absolute ethanol (5 mL) was stirred for the stipulated time.

The reaction progress was monitored using TLC; after completion of the reaction, the reaction mixture was cooled at room temperature, then crushed ice was used to get a solid product. The obtained solid was purified via column chromatography over silica gel (60–120 mesh) to afford a pure product.

6-chloro-2(1,3-diphenyl-1H-pyrazol-4-yl)-4-phenyl-1,2-dihydroquinazoline: **4a**.

Yellow solid; m.p. 160–162 °C; ^1^H NMR (400 MHz, CDCl_3_) δ 8.19 (s, 1H), 7.90 (t, J = 1.2 Hz, 2H), 7.75 (t, J = 1.2 Hz, 2H), 7.63–7.61 (m, 2H), 7.50–7.49 (m, 5H), 7.47–7.43 (m, 3H), 7.31 (t, J = 7.6 Hz, 1H), 7.25–7.22 (m, 2H), 6.62 (d, J = 8.4 Hz, 1H), 6.12 (s, 1H), 4.41 (s, 1H); ^13^C NMR (100 MHz, CDCl_3_) δ 165.1, 150.9, 145.5, 139.9, 137.4, 133.0, 132.6, 129.9, 129.4, 128.7, 128.4, 128.3, 128.2, 127.7, 126.5, 123.3, 123.2, 119.1, 119.1, 115.9, 65.0; HRMS: *m*/*z* calcd. For C_29_H_21_ClN_4_, 460.1455 found 460.1452.

2-(3-(4-bromophenyl)-1-phenyl-1H-pyrazol-4-yl)-6-chloro-4-phenyl-1,2-dihydroquinazoline: **4b**.

Yellow solid; m.p. 190–191 °C; ^1^H NMR (400 MHz, CDCl_3_) δ 8.06 (s, 1H), 7.71 (d, J = 8.8 Hz, 2H), 7.62 (d, J = 7.6 Hz, 2H), 7.52 (t, J = 8.4 Hz, 4H), 7.39–7.33 (m, 5H), 7.22–7.12 (m, 3H), 6.54 (d, J = 8 Hz, 1H), 5.97 (s, 1H), 4.25 (s, 1H); ^13^C NMR (100 MHz, CDCl_3_) δ 165.1. 149.8, 145.3, 139.8, 137.3, 132.7, 132.0, 129.9, 129.8, 129.4, 129.1, 128.4, 128.4, 127.8, 126.7, 123.5, 123.3, 122.5, 119.1, 115.9, 65.1; HRMS: *m*/*z* calcd. For C_29_H_20_BrClN_4_, 538.0560 found 538.0557.

6-chloro2-(3-(4-nitrophenyl)-1-phenyl-1H-pyrazol-4-yl)-4-phenyl-1,2-dihydroquinazoline: **4c**.

Yellow solid; m.p. 200–202 °C; ^1^H NMR (400 MHz, CDCl_3_) δ 8.33 (d, J = 6.8 Hz, 2H), 8.21 (t, J = 6 Hz, 3H), 7.74 (t, J = 8 Hz, 2H), 7.60–7.57 (m, 2H), 7.52–7.46 (m, 5H), 7.36 (t, J = 7.6 Hz, 1H), 7.30–7.27 (m, 1H), 7.25 (d, J = 2.4 Hz, 1H), 6.71 (d, J = 8.4 Hz, 1H), 6.12 (s, 1H), 4.41 (s, 1H); ^13^C NMR (100 MHz, CDCl_3_) δ 165.3, 148.6, 147.3, 145.2, 139.6, 137.1, 132.9, 130.1, 129.9, 129.5, 129.1, 128.8, 128.7, 128.6, 128.5, 128.1, 127.1, 124.1, 123.8, 119.2, 119.1, 116.0, 65.1; HRMS:*m*/*z* calcd. For C_29_H_20_ClN_5_O_2_, 505.9620 found 505.9617.

6-chloro2-(3-(4-methoxyphenyl)-1-phenyl-1H-pyrazol-4-yl)-4-phenyl-1,2-dihydroquinazoline: **4d**.

Yellow solid; m.p. 180–182 °C; ^1^H NMR (400 MHz, CDCl_3_) δ 8.07 (s, 1H), 7.73 (d, J = 6.8 Hz, 2H), 7.65 (d, J = 8 Hz, 2H), 7.53 (d, J = 7.2 Hz, 2H), 7.40 (d, J = 6.4 Hz, 3H), 7.37 (t, J = 8 Hz, 2H), 7.18–7.12 (m, 3H), 6.94 (d, J = 7.2 Hz, 2H), 6.52 (d, J = 8.8 Hz, 1H), 6.03 (s, 1H), 4.25 (s, 1H), 3.79 (s, 3H); ^13^C NMR (100 MHz, CDCl_3_) δ 165.0, 159.8, 145.5, 132.6, 129.8, 129.5, 129.3, 129.1, 128.4, 127.5, 126.4, 125.6, 122.8, 119.0, 115.8, 114.2, 65.1, 55.3; HRMS:*m*/*z* calcd. For C_30_H_23_ClN_4_O, 490.9910 found 490.9908.

2-(1,3-diphenyl-1H-pyrazol-4-yl)-6-nitro-4-phenyl-1,2-dihydroquinazoline: **4e**.

Yellow solid; m.p. 206–208 °C; ^1^H NMR (400 MHz, CDCl_3_) δ 8.11 (s, 2H), 8.05 (s, 1H), 7.83–7.80 (m, 2H), 7.67 (t, J = 1.2 Hz, 2H), 7.56–7.54 (m, 2H), 7.51–7.45 (m, 5H), 7.43–7.38 (m, 3H), 7.28 (t, J = 7.6 Hz, 1H), 6.55 (d, J = 8.8 Hz, 1H), 6.38 (s, 1H), 5.15 (s, 1H); ^13^C NMR (100 MHz, CDCl_3_) δ 164.3, 151.3, 150.7, 139.7, 138.6, 136.6, 132.7, 130.3, 129.4, 128.9, 128.7, 128.5, 128.2, 127.5, 126.8, 125.3, 122.8, 119.1, 115.3, 113.7, 65.0; HRMS:*m*/*z* calcd. For C_29_H_21_N_5_O_2_, 471.1695 found 471.1692.

2-(3-(4-bromophenyl)-1-phenyl-1H-pyrazol-4-yl)-6-nitro-4-phenyl-1,2-dihydroquinazoline: **4f**.

Yellow solid; m.p. 220–221 °C; ^1^H NMR (400 MHz, CDCl_3_) δ 8.05 (d, J = 3.2 Hz 2H), 7.98 (s, 1H), 7.68 (d, J = 7.6 Hz, 2H), 7.60 (d, J = 7.6 Hz, 2H), 7.52 (d, J = 7.6 Hz, 2H), 7.47–7.40 (m, 5H), 7.37 (t, J = 8 Hz, 2), 7.23–7.18 (m, 1H), 6.53 (d, J = 9.2 Hz, 1H), 6.30 (s, 1H), 5.01 (s, 1H); ^13^C NMR (100 MHz, CDCl_3_) δ 164.3, 151.1, 149.6, 139.6, 138.8, 136.5, 131.9, 131.7, 130.4, 129.8, 128.8, 128.7, 127.6, 127.0, 125.3, 122.9, 122.7, 119.2, 115.4, 113.7, 65.1; HRMS:*m*/*z* calcd. For C_29_H_20_BrN_5_O_2_, 549.0800 found 549.0802.

2-(3-(4-chlorophenyl)-1-phenyl-1H-pyrazol-4-yl)-6-nitro-4-phenyl-1,2-dihydroquinazoline: **4g**.

Yellow solid; m.p. 214–216 °C; ^1^H NMR (400 MHz, CDCl_3_) δ 8.05 (d, J = 3.2 Hz 2H), 7.98 (s, 1H), 7.74 (d, J = 7.6 Hz, 2H), 7.60 (d, J = 7.6 Hz, 2H), 7.47–7.39 (m, 5H), 7.36 (t, J = 7.2 Hz, 4H), 7.23–7.18 (m, 1H), 6.52 (d. J = 7.6 Hz, 1H), 6.31 (s, 1H), 5.01 (s, 1H); ^13^C NMR (100 MHz, CDCl_3_) δ 164.3, 151.1, 149.5, 139.6, 138.8, 136.5, 134.5, 131.2, 130.3, 129.5, 129.5, 129.0, 128.9, 128.8, 128.7, 127.6, 126.9, 125.3, 122.9, 119.2, 115.4, 113.7, 65.1; HRMS:*m*/*z* calcd. For C_29_H_20_ClN_5_O_2_, 505.9620 found 505.9618.

2-(3-(4-methoxyphenyl)-1-phenyl-1H-pyrazol-4-yl)-6-nitro-4-phenyl-1,2-dihydroquinazoline: **4h**.

Yellow solid; m.p. 196–198 °C; ^1^H NMR (400 MHz, CDCl_3_) δ 8.05 (d, J = 3.2 Hz 2H), 7.97 (s, 1H), 7.69 (d, J = 7.2 Hz, 2H), 7.60 (d, J = 8 Hz, 2H), 7.50 (d, J = 7.2 Hz, 2H), 7.44 (t, J = 6.8 Hz, 3H), 7.35 (t, J = 7.6 Hz, 2H), 7.21 (t, J = 9.2 Hz, 1H), 6.93 (d, J = 7.6 Hz 2H), 6.49 (d, J = 8.8 Hz, 1H), 6.33 (s, 1H), 5.03 (s, 1H), 3.78 (s, 3H); ^13^C NMR (100 MHz, CDCl_3_) δ 164.3, 159.9, 151.2, 150.5, 139.8, 138.7, 136.7, 130.3, 129.5, 129.4, 128.9, 128.7, 128.7, 127.4, 126.6, 125.3, 122.5, 119.1, 115.4, 114.3, 113.7, 65.2, 55.3; HRMS:*m*/*z* calcd. For C_30_H_23_N_5_O_3_, 500.1801.

4-((4-(6-chloro-4-phenyl-1,2-dihydroquinazolin-2-yl)-3-methyl-1-phenyl-1H-pyrazol -5-yl)oxy)benzonitrile: **4i**.

Yellow solid; m.p. 208–210 °C; ^1^H NMR (400 MHz, CDCl_3_) δ 7.53 (t, J = 1.6 Hz, 2H), 7.51–7.46 (m, 1H), 7.44–7.41 (m, 4H), 7.39–7.33 (m, 4H), 7.28 (t, J = 4 Hz, 1H), 7.17–7.15 (m, 1H), 7.00 (d, J = 2.4 Hz, 1H), 6.94 (d, J = 8.8 Hz, 2H), 6.56 (d, J = 8.4 Hz, 1H), 5.93 (s, 1H), 4.16 (s, 1H), 2.47 (s,3H); ^13^C NMR (100 MHz, CDCl_3_) δ 165.1, 159.8, 148.4, 145.2, 145.0, 137.5, 137.0, 134.0, 132.5, 129.8, 129.2, 128.7, 128.3, 128.1, 127.4, 123.1, 122.4, 118.3, 118.3, 116.4, 115.5, 109.3, 107.0, 64.5, 14.1; HRMS:*m*/*z* calcd. For C_31_H_22_ClN_5_O, 516.0010 found 516.0008.

6-chloro-2-(5-(4-chlorophenoxy)-3-methyl-1-phenyl-1H-pyrazol-4-yl)-4-phenyl-1,2- dihydroquinazoline: **4j**.

Yellow solid; m.p. 201–202 °C; ^1^H NMR (400 MHz, CDCl_3_) δ 7.57–7.55 (m, 2H), 7.44 (s, 5H), 7.38–7.34 (m, 2H), 7.28 (t, J = 4.4 Hz, 1H), 7.17–7.14 (m, 3H), 7.04 (s, 1H), 6.86–6.83 (m, 2H), 6.54–6.51 (m, 1H), 5.91 (s, 1H), 4.11 (s, 1H), 2.46 (s,3H); ^13^C NMR (100 MHz, CDCl_3_) δ 165.2, 155.6, 148.6, 146.0, 145.3, 137.8, 137.3, 132.4, 129.6, 129.1, 128.7, 128.4, 128.3, 128.2, 127.0, 123.0, 122.3, 118.3, 116.8, 115.4, 108.9, 64.6; HRMS:*m*/*z* calcd. For C_30_H_22_Cl_2_N_4_O, 525.4330 found 525.4328.

2-(5-(4-bromophenoxy)-3-methyl-1-phenyl-1H-pyrazol-4-yl)-6-chloro-4-phenyl-1,2- dihydroquinazoline: **4k**.

Yellow solid; m.p. 198–200 °C; ^1^H NMR (400 MHz, CDCl_3_) δ 7.46 (d, J = 7.6 Hz, 2H), 7.36 (t, J = 9.2 Hz, 5H), 7.27 (t, J = 7.2 Hz, 2H), 7.19–7.13 (m, 3H), 7.07 (d, J = 8.4 Hz, 1H), 6.93 (s, 1H), 6.69 (d, J = 7.2 Hz, 2H), 6.42 (d, J = 8Hz, 1H), 5.81 (s, 1H), 3.98 (s, 1H), 2.35 (s, 3H); ^13^C NMR (100 MHz, CDCl_3_) δ 165.1, 156.2, 148.5, 145.9, 145.3, 137.8, 132.6, 132.4, 129.6, 129.1, 128.7, 128.3, 128.2, 127.1, 123.0, 122.3, 118.3, 117.3, 115.9, 115.4, 108.9, 64.6, 14.3; HRMS:*m*/*z* calcd. For C_30_H_22_BrClN_4_O, 568.0666 found 568.0662.

6-chloro-2-(5-(2,4-dichlorophenoxy)-3-methyl-1-phenyl-1H-pyrazol-4-yl)-1,2-dihydroquinazoline: **4l**.

Yellow solid; m.p. 182–183 °C; ^1^H NMR (400 MHz, CDCl_3_) δ 7.61 (d, J = 7.2 Hz, 2H), 7.43 (s, 5H), 7.39 (t, J = 7.2 Hz, 2H), 7.28–7.23 (m, 2H), 7.16 (d, J = 8.8 Hz, 1H), 7.02 (s, 1H), 6.94 (d, J = 8.8 Hz, 1H), 6.72 (d, J = 8.8 Hz, 1H), 6.56 (d, J = 8.4 Hz, 1H), 5.94 (s, 1H), 4.23 (s, 1H), 2.47 (s, 3H); ^13^C NMR (100 MHz, CDCl_3_) δ 165.1, 151.2, 148.4, 145.2, 137.6, 137.2, 132.5, 130.1, 129.6, 129.1, 128.8, 128.5, 128.3, 128.1, 127.6, 127.2, 123.0, 123.0, 122.2, 118.2, 116.4, 115.5, 109.1, 64.6, 14.2; HRMS:*m*/*z* calcd. For C_30_H_21_Cl_3_N_4_O, 558.0781 found 558.0779.

6-chloro-2-(3-methyl-5-(4-nitrophenoxy)-1-phenyl-1H-pyrazol-4-yl)-4-phenyl-1,2-dihydroquinazoline: **4m**.

Yellow solid; m.p. 190–191 °C; ^1^H NMR (400 MHz, CDCl_3_) δ 8.00 (d, J = 7.6 Hz, 2H), 7.55 (d, J = 7.6 Hz, 2H), 7.46–7.34 (m, 6H), 7.28–7.24 (m, 2H), 7.17 (d, J = 8.8 Hz, 1H), 6.99 (s, 1H), 6.93 (d, J = 7.6 Hz, 2H), 6.56 (d, J = 8.4 Hz, 1H), 5.95 (s, 1H), 4.16 (s, 1H), 2.48 (s, 3H); ^13^C NMR (100 MHz, CDCl_3_) δ 165.0, 161.3, 148.4, 145.1, 143.3, 137.5, 137.0, 132.4, 129.8, 129.2, 128.6, 128.2, 128.1, 127.4, 125.6, 123.2, 122.4, 118.3, 115.9, 115.5, 109.4, 64.6, 14.0; HRMS:*m*/*z* calcd. For C_30_H_22_ClN_5_O_3_, 535.1411 found 535.1408.

6-chloro-2-(5-(4-methoxyphenoxy)-3-methyl-1-phenyl-1H-pyrazol-4-yl)-4-phenyl-1,2-dihydroquinazoline: **4n**.

Yellow solid; m.p. 184–185 °C; ^1^H NMR (400 MHz, CDCl_3_) δ 7.58 (d, J = 7.6 Hz, 2H), 7.52 (t, J = 5.2 Hz, 1H), 7.45–7.39 (m, 5H), 7.35 (t, J = 7.6 Hz, 2H), 7.23 (t, J = 7.2 Hz, 1H), 7.13–7.10 (m, 1H), 7.01 (d, J = 2.4 Hz, 1H), 6.84 (d, 2H), 6.73–6.70 (m, 2H), 6.46 (d, J = 8.4 Hz, 1H), 5.86 (s, 1H), 3.71 (s, 3H), 2.41 (s, 3H); ^13^C NMR (100 MHz, CDCl_3_) δ 165.1, 155.5, 151.2, 148.6, 147.0, 145.6, 138.0, 137.4, 132.3, 130.0, 129.5, 129.0, 128.8, 128.2, 126.8, 122.8, 122.3, 116.5, 114.7, 64.6, 55.6, 14.6; HRMS:*m*/*z* calcd. For C_31_H_25_ClN_4_O_2_, 521.0170 found 521.0169.

4-((3-methyl-4-(6-nitro-4-phenyl-1,2-dihydroquinazolin-2-yl)-1-phenyl-1H-pyrazol- 5-yl)oxy)benzonitrile: **4o**.

Yellow solid; m.p.178–180 °C; ^1^H NMR (400 MHz, CDCl_3_) δ 7.98 (d, J = 8.8 Hz, 1H), 7.85 (s, 1H), 7.42 (d, J = 8Hz, 3H), 7.37 (t, J = 6.8 Hz, 2H), 7.31–7.23 (m, 6H), 7.18 (t, J = 8.4 Hz, 1H), 6.79 (d, J = 7.2 Hz, 2H), 6.44 (d, J = 8.8 Hz, 1H), 6.18 (s, 1H), 4.94 (s, 1H), 2.34 (s, 3H); ^13^C NMR (100 MHz, CDCl_3_) δ 164.1, 159.6, 151.0, 148.0, 145.0, 138.4, 137.3, 136.3, 134.0, 130.2, 129.3, 128.7, 128.6, 128.4, 127.6, 125.1, 122.3, 118.0, 116.2, 114.2, 113.3, 109.5, 107.2, 64.7, 13.8; HRMS:*m*/*z* calcd. For C_31_H_22_N_6_O_3_, 526.5560 found 526.5558.

### 2.6. Antioxidant Activity of ZnO-NPs

The standard DPPH method was used to evaluate the antioxidant activity of the synthesized ZnO nanoparticles. The activity was measured for various concentrations of the ZnO nanoparticles, which ranged from 0.001 to 0.004 mM (test solution—3 mL added to 1 mL of 0.1 mM methanolic DPPH). The same experiment was carried out with ascorbic acid, which is used as standard. The blank solution was prepared by adding 1 mL of DPPH methanolic solution. These solutions were incubated for 30 min in a dark room, then the absorbance values of the standard and test solutions were recorded by using the UV-Visible spectrophotometer at 517 nm.

### 2.7. General Procedure for Assessing the Antioxidant Activity of 1,2-Dihydroquinazolines with the DPPH Method

The free-radical scavenging activity of synthesized 1,2-dihydroquinazoline derivatives (**4a**–**o**) was assessed using a standard DPPH method [46]. Antioxidants reduce the damage caused by free radicals. These antioxidants are generally hydrogen donors; they donate hydrogen to reduce the stable DPPH, causing it to change color i.e., from deep violet to yellow. The change in color of the compounds and the standard was measured at 517 nm with the UV-Visible spectrophotometer. The test solutions (**4a**–**o**) were subjected to serial dilution using a methanol solvent, ranging from 0.001 to 0.004 mM. At this point, 1 mL of 0.1 mM methanolic DPPH solution was mixed with 3 mL of the test solution, then these solutions were incubated in the dark for about 30 min. The absorbance of the control solution (without sample) and test solutions was measured at 517 nm. In this DPPH method, ascorbic acid is used as standard. The absorbance values of the sample solutions were measured in triplicate. The IC_50_ values of the test samples and standard were calculated using the inhibition formula.

The percentage of inhibition was calculated using the following formula:

(1) $$\%\;\mathrm{of}\;\mathrm{inhibition}=\frac{\mathrm{Ac}-\mathrm{As}}{\mathrm{Ac}\prime }\;\times \;100$$

where Ac represents the absorbance of the control sample and As represents the absorbance of the test sample.

## 3. Results and Discussion

In the current investigation, ZnO nanoparticles were synthesized using sunflower-leaf aqueous extract solution, which was used as a solvent instead of organic solvents. Rapid green-patch biosynthesis with ZnO nanoparticles using sunflower leaf extract has been investigated previously as a simple, easy, cost-efficient, non-toxic, eco-friendly, and efficient method for achieving synthesis in a short time period. Plant extracts may act both as a reducing agent and as a stabilizing agent in the synthesis of ZnO nanoparticles.

**Figure 3:**
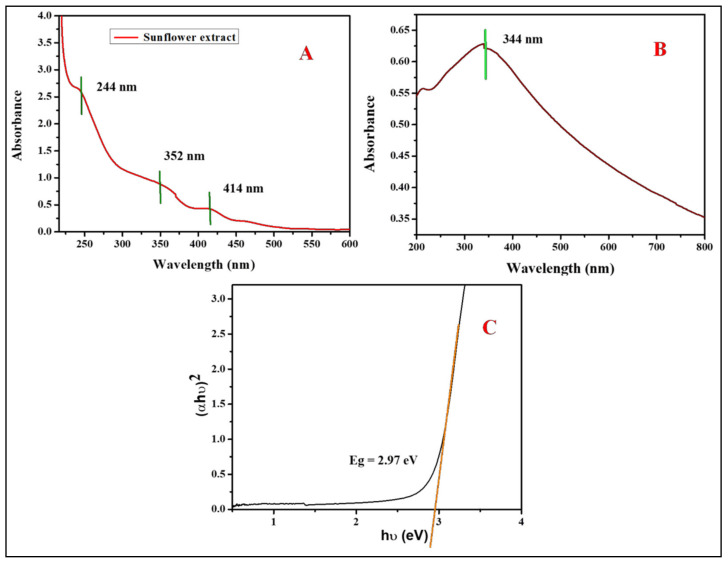
UV-Visible analysis of sunflower-leaf aqueous extract mediated by synthesized ZnO nanoparticles: (**A**) UV-Visible spectrum of sunflower-leaf aqueous extract; (**B**) UV-Visible absorbance spectrum of ZnO nanoparticles; (**C**) UV-DRS spectrum of ZnO nanoparticles.

Figure 3A shows the UV-Visible spectrum of the sunflower leaf extract. Three absorption bands can be observed. Band I (λmax 244 nm) and band II (λmax 352 nm), both bands in the UV region, might be attributed to (π→π*) transitions, while Band III (λmax 414 nm) might correspond to (n→π*) transitions. Previous reports [47] showed two absorption bands for the *Withania coagulans* leaf extract at 200–250 and 300–350 nm. The optical properties of the extract were determined using UV-Visible spectroscopy. Figure 3B displays the characteristic absorption spectrum of ZnO-NPs. Earlier reports [48] showed that the UV-Visible absorption band of ZnO nanoparticles ranges between 300 and 400 nm. The formation of the absorption band of ZnO-NPs was noticed at λmax 344 nm. The absorption coefficient for ZnO-NPs was determined using the following equation:

(2) E_g_ = (hʋ) − (αhʋ)^1/2^

where hʋ is the photon energy, α is the optical absorption coefficient and E_g_ represents the band gap energy.

Figure 3C shows the optical band gap plot. The band gap energy was measured by plotting (αhʋ)^1/2^ of ZnO vs. the photon energy (hʋ). Therefore, the band gap energy value of ZnO-NPs is 2.97 eV. The generated results are strongly analogous to those mentioned by Spada et al. [49].

**Figure 4:**
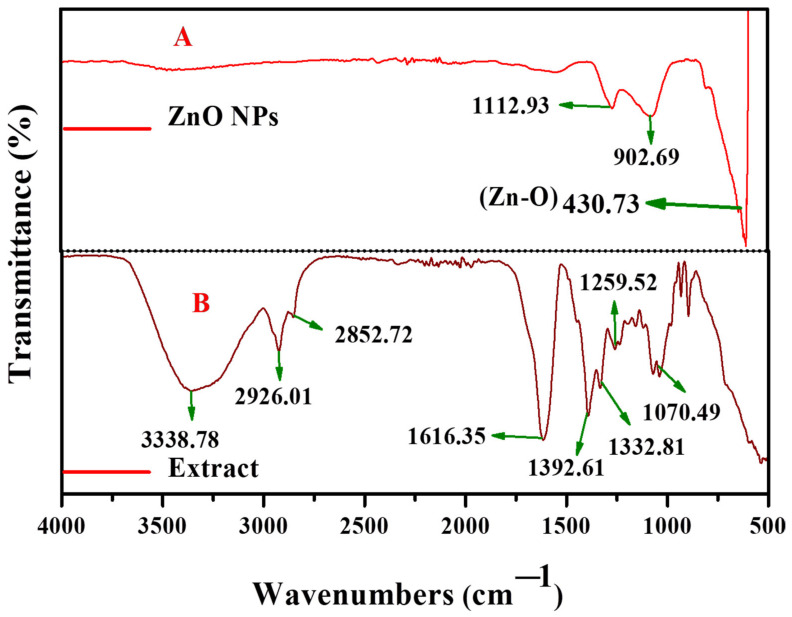
FT-IR spectroscopy of ZnO nanoparticles (**A**) and sunflower leaf extract (**B**).

FTIR analysis identified the functional groups in the plant extract and the capping and reducing agent’s major role in the formation of ZnO nanoparticles. Figure 4A,B shows the FTIR spectrum of ZnO nanoparticles and the plant extract; in the plant extract, the peak at 3338 cm^−1^ corresponds to the (–O–H) hydroxyl group of stretching vibrations. The peaks at 2926 and 2852 cm^−1^ were attributed to the symmetric and asymmetric –CH stretching vibrations of the methyl group, respectively. A strong peak at 1616 cm^−1^ represented the carbonyl group (–C=O) stretching vibration. In a similar way, prior reports [50] showed *Leucaena leucocephala* bark extract to have FTIR bands of 2918 and 2850 cm^−1^, corresponding to the symmetric and asymmetric methyl and methylene groups’ stretching vibration, respectively. The peak located at 1392 cm^−1^ it may be assigned to the carbon and oxygen (–C–O) stretching vibration. In the present spectrum, there is a strong absorption band at around 1332 cm^−1^ which was attributed to carbon and oxygen and carbon (–C–O–C–) stretching vibrations. The peak arising at 1259 cm^−1^ may be due to carbon and nitrogen (–C–N) stretching vibrations. The peaks below 1000 cm^−1^ correspond to the bending vibration of polyphenols was represented Figure 4B.

In the FTIR spectroscopy of ZnO nanoparticles, the Spectrum bands were noted at around 1112 cm^−1^, corresponding to the carbon and oxygen and carbon (–C–O–C–) stretching vibrations. The peak showed a strong absorption band at around 430 cm^−1^ representing the metal and oxygen (Zn–O) stretching vibration was represented Figure 4A.

**Figure 5:**
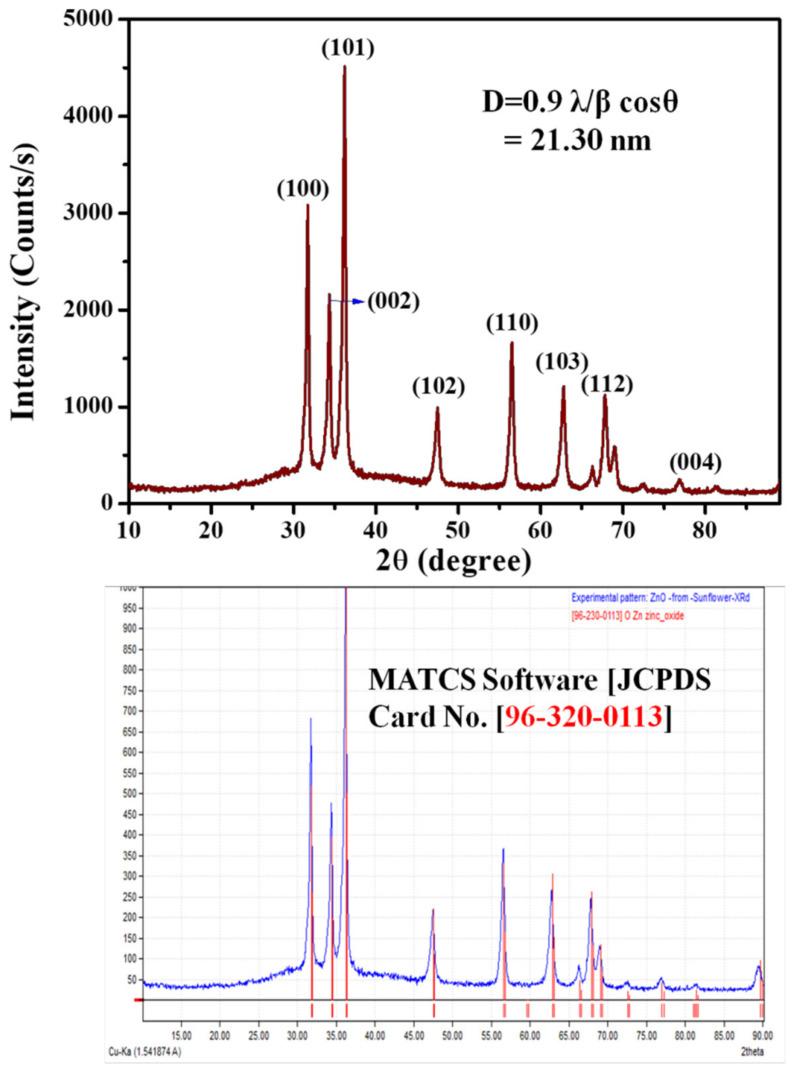
The synthesized ZnO NPs of XRD and standard JCPDS XRD data.

The current reported results resemble those of Pudukudy et al. [51], where a metal and oxygen stretching vibration was noted at 488 cm^−1^. The XRD technique is used for the phase determination of the crystal structures of the nanoparticles, as shown in Figure 5. The XRD analysis of the synthesized particles shows characteristic diffraction peaks at 2θ of 31.82, 34.41, 36.32, 47.55, 56.58, 63.02, 67.93, 76.98, which were assigned to (100), (002), (101), (102), (110), (103), (112) and (004) planes, respectively. From the analysis by XRD, a hexagonal structure for the ZnO nanoparticles prepared from the sunflower leaf extract was suggested. The hexagonal structure of ZnO was obtained. It was then confirmed by comparison with the data provided by MATCH! software (JCPDS card No. 96-320-0113) as shown in (Figure 5), and the cell parameter of the synthesized particle is a = b = 3.2494 Å and c = 5.2054 Å.

All the diffraction peaks correspond to a typical hexagonal structure and no other phase was observed [52]. The average crystallite size of the ZnO nanoparticles was calculated using the Scherrer formula, D = 0.9 λ/β cos θ, where λ is the wavelength of X-ray radiation and β is the full width at half maximum (FWHM) of the peaks at the diffracting angle θ (Table 2).

**Figure 6:**
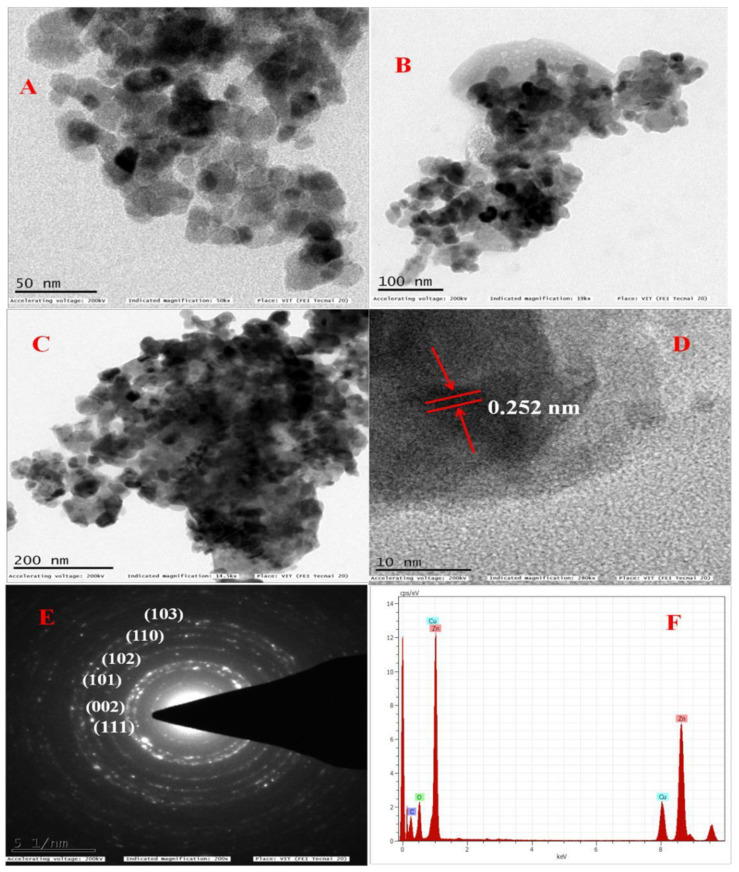
TEM images showing the ZnO-NPs at different magnifications: (**A**) (50 nm), (**B**) (100 nm), (**C**) (200 nm); (**D**) represents the d-spacing, (**E**) denotes the SEAD pattern of polycrystalline, (**F**) rep-resents the EDAX analysis of the ZnO-NPs.

Table 2 shows the geometrical parameters of the ZnO nanoparticles. The average crystallite size was calculated using the Scherrer formula, and the size was found to be 20.46 nm. The size and morphology of the synthesized ZnO nanoparticles were analyzed using high-resolution transmission electron microscopy (HR-TEM). Figure 6A–F shows the TEM images of the ZnO nanoparticles at different scale magnifications, for instance, 50 nm, 100 nm, and 200 nm. Figure 6A,E shows the histogram of the ZnO nanoparticles; it was observed that most of the nanoparticles are spherical in nature, without any agglomeration, and the size ranged between 12 and 25 nm. The average particle size was calculated to be around 17.25 nm. Figure 6D represents the d-spacing of the ZnO nanoparticles; this was found to be 0.252 nm, which is in good agreement with the XRD result.

Figure 6E reveals the selective area electron diffraction pattern (SAED), which gives clear information about the crystallinity of the ZnO nanoparticles. The bright spots indicate the crystalline nature, whereas the spots in the form of circular rings are due to the polycrystalline nature of ZnO nanoparticles, established from the obtained results. The sizes and lattice planes of the ZnO nanoparticles are in good agreement with the XRD data. Sangeetha et al. [53] reported a similar result. Figure 6F shows the characteristic elemental composition of zinc oxide nanoparticles, obtained from the energy-dispersive X-ray spectroscopy (EDAX) study. This suggests the zinc and oxygen elements of atomic percentage and atomic weight percentage, such that zinc (wt % 51.57, atomic wt % 21.69), oxygen (wt % 7.33, atomic wt % 12.60), and the remaining atomic peaks in the SAED pattern are due to the copper grid, which confirms the purity of the ZnO nanoparticles. From the above data, we can conclude that the obtained nanoparticles are pure and polycrystalline in nature.

**Figure 7:**
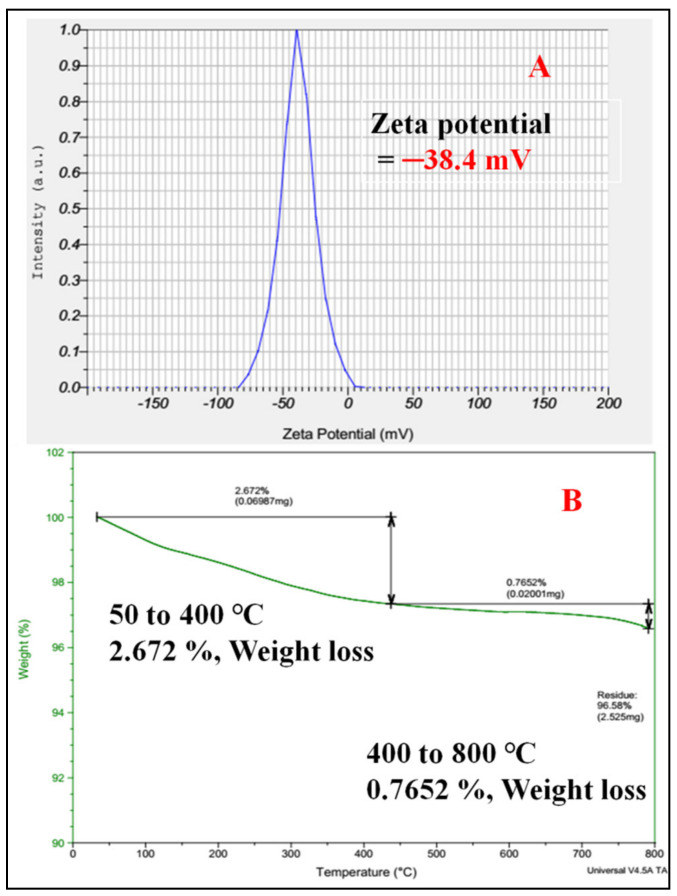
Stability analysis of ZnO-NPs: (**A**) colloidal stability; (**B**) thermal stability.

Zeta potential is a key indicator of the stability of the colloidal solution. Figure 7A describes the colloidal solution of ZnO nanoparticles, the stability of the nanoparticles being expressed in terms of zeta value, which was found to be −38.4 mV. This is a direct result, based on the mobility of the charged particles. The results suggest that the good stability of the ZnO nanoparticles, shown in the higher zeta potential value, may be describing the higher electrical charge on the surface of the particles [54]. The thermal stability of the ZnO nanoparticles was studied using thermogravimetric analysis (TGA). Figure 7B shows the thermal stability of the ZnO nanoparticles. From the total TGA plot, the weight loss of the compound was as follows: (i) the 2.672% initial weight loss of the compound at a temperature of 50–400 °C was due to moisture in the compound; (ii) 0.7652% of the weight loss of the compound at a temperature of 400–800 °C may be attributed to the removal of volatile compounds. In the range of 400–800 °C, a very low weight loss was seen in the compound and no further weight loss was observed, so we concluded that the rest of the content is pure and has a crystalline nature [55].

### 3.1. Catalytic Activity of ZnO Nanoparticles

**Figure 8:**
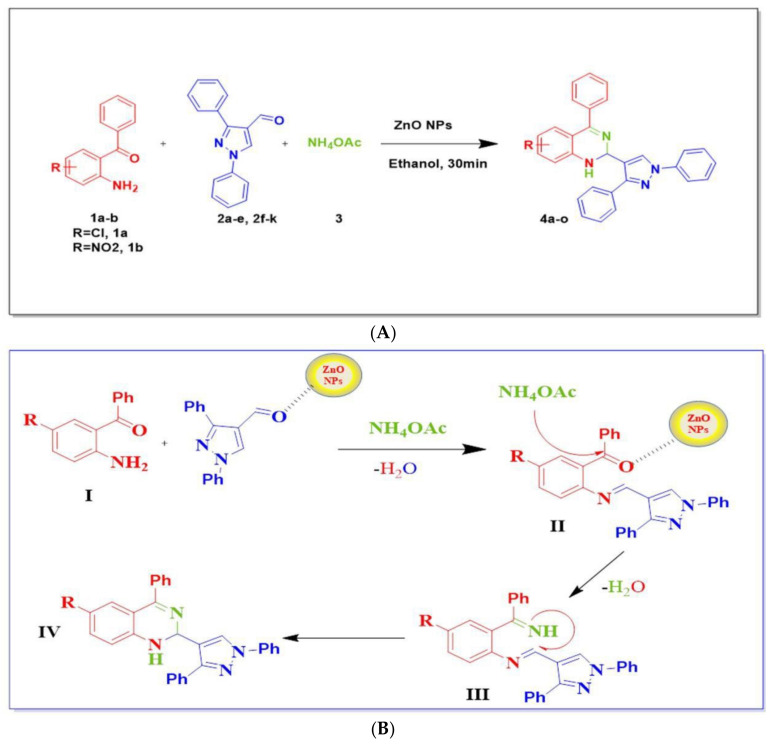
(**A**) Synthesis of 2-(1,3-diphenyl-1H-pyrazol-4-yl)-4-phenyl-1,2-dihydroquinazoline derivatives. (**B**) A plausible mechanism for the synthesis of 1,2-dihydroquinazolines by ZnO nanoparticles.

The synthetic pathway for the preparation of 1,2-dihydroquinazoline derivatives (Figure 8A) by the one-pot three-component reaction of 2-amino 5-chloro benzophenone (**1a**) (1eq), 1,3-diphenylpyrazol4-carbaldehydes (**2a**) (1eq), and ammonium acetate (3eq) was added to 5 mL of ethanol and stirred at room temperature for 12 h. We found that there was no progress of the reaction. Then, the same reaction was carried out under reflux conditions for 6 h, after which we obtained 20% of the desired product (Figure 8B) via the formation of imine intermediates.

The reaction was performed without any catalyst, affording only traces of the product, which indicates the necessity of using the catalyst to improve product yields. Then, we assessed the reaction by using different catalysts; with the addition of a piperidine catalyst, the desired product was obtained at 53% yield, while later, the addition of DMAP to the reaction mixture at reflux conditions resulted in a 62% yield. Later, we performed the reactions using base catalysts like KOH and TEA under reflux conditions; the resulting product yields were increased moderately. When we used the radical initiator catalyst (AIBN), the required 1,2-dihydroquinazoline product was formed with a lower yield; the catalyst does not show any significant effect on the synthesis of product **4a**. Later, we conducted trials with acid catalysts like PTSA and H_3_PO_4_ and achieved the desired product with good yields (Table 3). A good yield was achieved by using a heterogeneous catalyst (MK-10) in ethanol solvent under reflux conditions. Among all the trials examined, we found that the above catalysts were not suitable for achieving good yields in the formation of 1,2 dihydro quinazolines. To synthesize product **4a**, we used the ZnO nanoparticles under reflux conditions and an excellent yield was obtained, i.e., 95%. A product yield of 90% was achieved by using 5 mol % of catalyst; when we increased the catalytic concentration, that is, from 5 mol % to 15 mol %, this afforded product **4a** at a 94% yield (Table 1). Similarly, 90% of the product yield was formed by using 20 mol % of catalyst. This reveals that increasing the catalyst loading percentage does not show any significant yield increase in terms of product formation. The same reaction was carried out using different solvents, like methanol, DMF, ethyl acetate, and water; the product was formed with good yields.

**Figure 9:**
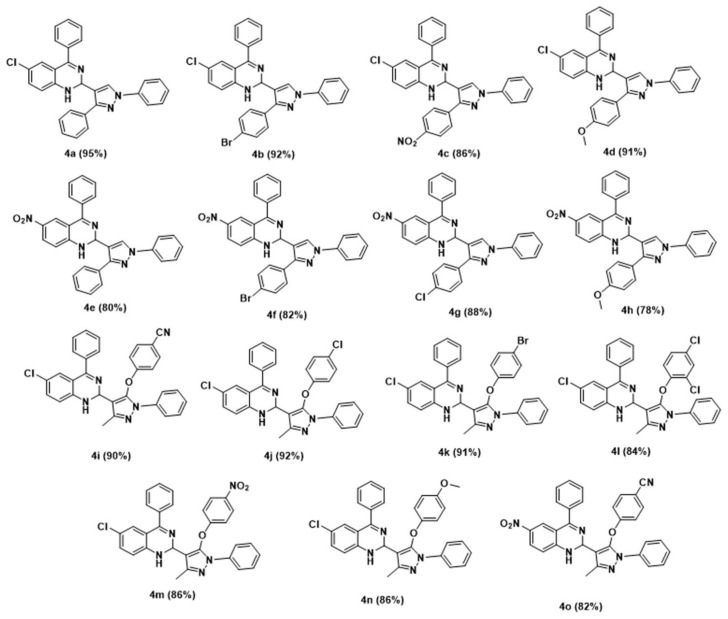
Diversity of the synthesized 1,2-dihydroquinazolines.

Thus, under optimized conditions, an overview of the reaction was investigated by employing several pyrazole 4-carbaldehydes, **2a**–**e**, and **2f**–**k**. The obtained results are depicted in Figure 9.

The scope of this transformation was expanded by the study of the reaction of 2-amino benzophenones **1a**–**b** substituted with 1,3-diphenylpyrazole-4-carbaldehydes (**2a**–**e**). It was observed that various functional groups play a significant role in achieving product yields. Generally, the substituted 2-aminobenzophenones reacted well, with several pyrazole aldehyde derivatives giving the corresponding products good to excellent yields. When 5-nitro-2-aminobenzophenone **1b** was reacted with 1,3-diphenyl-1H-pyrazole-4-carbaldehydes **2a**–**e** and 3-methyl-5-phenoxy-1-phenyl-1H-pyrazole-4-carbaldehydes **2f**–**k**, the reaction product was obtained at an excellent yield, but the reaction with 4-methoxy 1,3-diphenyl-1H-pyrazole-4-carbaldehyde **2d** afforded the product at a lower yield. When 5-nitro-2-aminobenzophenone was replaced with electron-withdrawing groups, such as chloro substituents, the 1,2-dihydroquinazoline derivatives were obtained in good yields.

### 3.2. Antioxidant Activity of ZnO Nanoparticles

The antioxidant activity of synthesized ZnO nanoparticles was examined and the percentage of inhibition and IC50 values were calculated; our results showed the effective radical scavenging activity of nanoparticles. The color variation, i.e., from blue to yellow, was observed with the addition of ZnO nanoparticles. At 517 nm, the absorbance decreased with an increase in the concentrations of ZnO nanoparticles. The synthesized nanoparticles acted as an electron donor that reacts with free radical molecules to change them to stable molecules.

**Figure 10:**
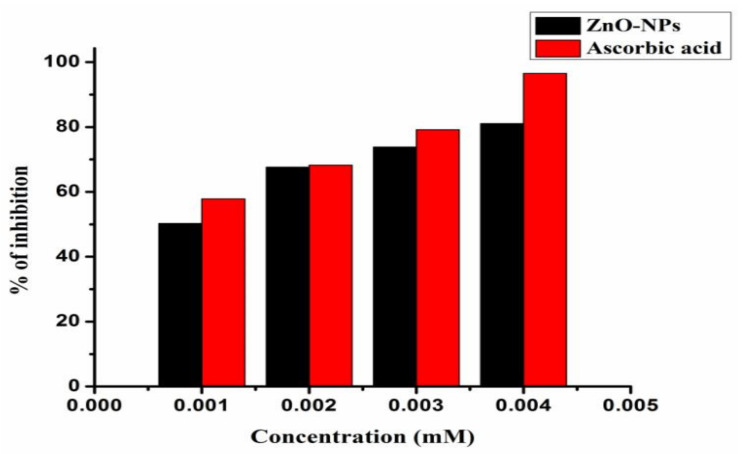
Antioxidant activity of ZnO nanoparticles, compared with standard ascorbic acid.

We have shown that activity increased with the increasing concentration of ZnO nanoparticles. The antioxidant activity of ZnO nanoparticles was described; their percentages of inhibition are shown in Figure 10. The nanoparticles showed a good percentage of inhibition at different concentrations (mM) and good IC_50_ values (0.769) against standard ascorbic acid (0.511). In a similar way, Narasaiah et al. [56] have reported the antioxidant activity of ZnO NPs obtained using agro-waste durva grass aqueous extract.

### 3.3. Antioxidant Activity of Synthesized 1,2-Dihydro Quinazolines

**Figure 11:**
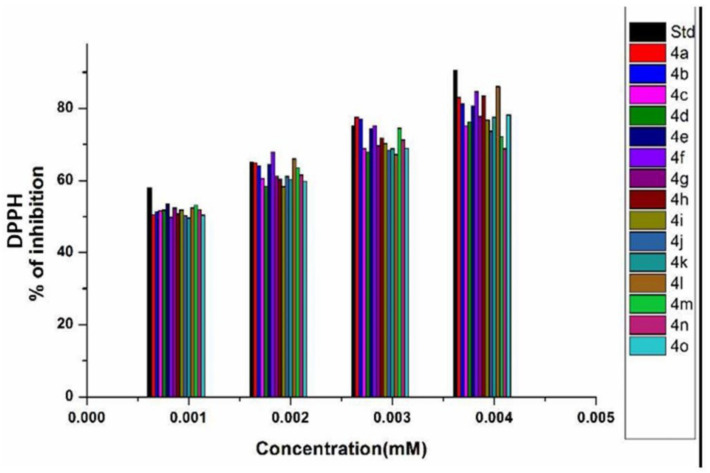
DPPH activity of the synthesized 1,2-dihydroquinazolines.

The DPPH values of methanolic solutions of 1,2-dihydroquinazoline derivatives were examined. From the results, we can assume that the scavenging effect of 1,2-dihydroquinazolines (**4a**–**o**) on DPPH radicals increased with increasing concentration; we measured the IC_50_ values and the percentage of inhibition for synthesized derivatives at different concentrations (Table 4). The obtained results (Figure 11) show that the 1,2-dihydroquinazolines were proven to demonstrate good scavenging properties compared with standard ascorbic acid.

## 4. Conclusions

In this study, we demonstrated that sunflower-leaf extract ZnO nanoparticles were found to be a good catalyst for the synthesis of 1,2-dihydroquinazolines analogs under normal conditions, giving an enhanced yield with a reduced time period. The synthesized compounds (**4a**–**o**) were screened for radical scavenging activity using a standard DPPH method. All the compounds showed a high percentage of inhibition compared to standard ascorbic acid.

## Figures and Tables

**Table 1:** Optimization of the catalyst (ZnO-NPs) loading.

Entry	Catalyst	Catalyst Loading (Mol%)	Yield (%) 4a
1	ZnO-NPs	05	90
2	ZnO-NPs	10	95
3	ZnO-NPs	15	94
4	ZnO-NPs	20	90

**Table 2:** The structure and geometric parameters of the ZnO nanoparticles.

2 Theta	FWHM Value	d-Spacing (Å)	Cos (θ)	Crystalline Size (nm)
31.61	0.35	2.83	0.96221	24.65
34.23	0.38	2.61	0.95574	22.86
36.16	0.42	2.48	0.91262	20.79
47.61	0.53	1.90	0.95062	17.12
56.64	0.48	1.62	0.88031	19.65
62.83	0.55	1.47	0.85345	17.69
Average size				20.46

**Table 3:** Optimization of the reaction conditions **4a**.

Entry	Catalyst	Catalyst Loading (Mol %)	Solvent	Temperature (°C)	Time (h)	Yield (%) 4a
1	---	---	Ethanol	RT	12	
2	---	---	Ethanol	Reflux	6	20
3	Piperidine	20	Ethanol	50	5	53
4	DMAP	20	Ethanol	Reflux	6	62
5	TEA	25	Ethanol	Reflux	5	60
6	KOH	20	Ethanol	Reflux	4	62
7	AIBN	20	Ethanol	Reflux	6	24
8	PTSA	20	Ethanol	Reflux	4	76
9	H_3_PO_4_	20	Ethanol	60	6	72
10	MK-10	20	Ethanol	Reflux	5	78
11	ZnO-NPs	10	Ethanol	Reflux	0.5	95
12	ZnO-NPs	10	Methanol	Reflux	1	91
13	ZnO-NPs	10	DMF	Reflux	2	72
14	ZnO-NPs	10	Ethyl acetate	60	2	79
15	ZnO-NPs	10	Water	Reflux	3	82

**Table 4:** Percentage of inhibition and IC_50_ values of compounds **4a**–**4o**.

Percentage of Inhibition at Different Concentrations (mM)						
Entry	Compound	0.001	0.002	0.003	0.004	IC_50_
1	**4a**	50.51	64.81	77.57	82.98	0.837
2	**4b**	51.15	64.04	77.06	81.18	0.794
3	**4c**	51.54	60.56	68.81	75.25	0.740
4	**4d**	51.80	58.37	67.91	76.28	0.870
5	**4e**	53.35	64.43	74.35	80.67	0.546
6	**4f**	49.74	67.91	75.25	84.66	0.842
7	**4g**	52.44	61.21	69.58	77.70	0.690
8	**4h**	50.77	60.30	71.77	83.37	0.988
9	**4i**	51.80	58.24	70.23	76.80	0.885
10	**4j**	50.25	61.08	68.29	73.71	0.823
11	**4k**	49.61	60.18	68.81	77.57	0.985
12	**4l**	52.44	65.97	67.26	86.08	0.902
13	**4m**	53.09	63.40	74.48	72.16	0.596
14	**4n**	51.93	61.46	71.26	68.81	0.715
15	**4o**	50.38	59.79	68.94	78.22	0.953
16	ZnO NPs	50.29	67.63	73.84	81.04	0.769
17	Ascorbic acid	57.98	64.94	75.12	90.59	0.511

## Data Availability

Data is contained within the article.
